# Silver(I) diaqua­nickel(II) *catena*-borodiphosphate(V) hydrate, (Ag_0.57_Ni_0.22_)Ni(H_2_O)_2_[BP_2_O_8_]·0.67H_2_O

**DOI:** 10.1107/S160053681102962X

**Published:** 2011-07-30

**Authors:** Hafid Zouihri, Mohamed Saadi, Boujemaa Jaber, Lahcen El Ammari

**Affiliations:** a Centre National pour la Recherche Scientifique et Technique, Division UATRS, Angle Allal AlFassi et Avenue des FAR, Hay Ryad, BP 8027, Rabat, Morocco; b Laboratoire de Chimie du Solide Appliquée, Faculté des Sciences, Université Mohammed V-Agdal, Avenue Ibn Batouta, BP 1014, Rabat, Morocco

## Abstract

The structure framework of the title compound, (Ag_0.57_Ni_0.22_)Ni(H_2_O)_2_[BP_2_O_8_]·0.67H_2_O, is the same as that of its recently published counterpart AgMg(H_2_O)_2_[BP_2_O_8_]·H_2_O. In the title structure, the Ag, Ni, B and one O atom are located on special positions (sites symmetry 2). The structure consists of infinite borophosphate helical [BP_2_O_8_]^3−^ ribbons, built up from alternate BO_4_ and PO_4_ tetra­hedra arranged around the 6_5_ screw axes. The vertex-sharing BO_4_ and PO_4_ tetra­hedra form a spiral ribbon of four-membered rings in which BO_4_ and PO_4_ groups alternate. The ribbons are connected through slightly distorted NiO_4_(H_2_O)_2_ octa­hedra, four O atoms of which belong to the phosphate groups. The resulting three-dimensional framework is characterized by hexa­gonal channels running along [001]. However, the main difference between the structures of these two compounds lies in the filling ratio of Wyckoff positions 6*a* and 6*b* in the tunnels. Indeed, in this work, the refinement of the occupancy rate of sites 6*a* and 6*b* shows that the first is occupied by water at 67% and the second is partially occupied by 56.6% of Ag and 21.6% of Ni. In the AgMg(H_2_O)_2_[BP_2_O_8_]·H_2_O structure, these two sites are completely occupied by H_2_O and Ag^+^, respectively. The title structure is stabilized by O—H⋯O hydrogen bonds between water mol­ecules and O atoms that are part of the helices.

## Related literature

For the isotypic Mg analogue, see: Zouihri *et al.* (2011 ▶); Menezes *et al.* (2008 ▶). For other borophosphates, see: Kniep *et al.* (1998 ▶); Ewald *et al.* (2007 ▶); Lin *et al.* (2008 ▶). For bond valence calculations see: Brown & Altermatt (1985 ▶).

## Experimental

### 

#### Crystal data

(Ag_0.57_Ni_0.22_)Ni(H_2_O)_2_[BP_2_O_8_]·0.67H_2_O
*M* *_r_* = 381.35
Hexagonal, 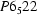

*a* = 9.3848 (6) Å
*c* = 15.8411 (18) Å
*V* = 1208.28 (18) Å^3^
*Z* = 6
Mo *K*α radiation
μ = 4.69 mm^−1^
*T* = 296 K
0.18 × 0.12 × 0.11 mm

#### Data collection

Bruker APEXII CCD detector diffractometer
Absorption correction: multi-scan (*SADABS*; Sheldrick, 1999 ▶) *T*_min_ = 0.705, *T*_max_ = 0.741
16974 measured reflections
2043 independent reflections
1912 reflections with *I* > 2σ(*I*)
*R*_int_ = 0.037

#### Refinement

*R*[*F* ^2^ > 2σ(*F* ^2^)] = 0.028
*wR*(*F* ^2^) = 0.071
*S* = 1.08
2043 reflections
77 parameters
1 restraint
H-atom parameters constrained
Δρ_max_ = 1.23 e Å^−3^
Δρ_min_ = −0.59 e Å^−3^
Absolute structure: Flack (1983 ▶), 779 Friedel pairs
Flack parameter: 0.006 (15)

### 

Data collection: *APEX2* (Bruker, 2005 ▶); cell refinement: *SAINT* (Bruker, 2005 ▶); data reduction: *SAINT*; program(s) used to solve structure: *SHELXS97* (Sheldrick, 2008 ▶); program(s) used to refine structure: *SHELXL97* (Sheldrick, 2008 ▶); molecular graphics: *ORTEP-3 for Windows* (Farrugia, 1997 ▶) and *DIAMOND* (Brandenburg, 2006 ▶); software used to prepare material for publication: *WinGX* (Farrugia, 1999 ▶).

## Supplementary Material

Crystal structure: contains datablock(s) I, global. DOI: 10.1107/S160053681102962X/br2172sup1.cif

Structure factors: contains datablock(s) I. DOI: 10.1107/S160053681102962X/br2172Isup2.hkl

Additional supplementary materials:  crystallographic information; 3D view; checkCIF report

## Figures and Tables

**Table 1:** Hydrogen-bond geometry (Å, °)

*D*—H⋯*A*	*D*—H	H⋯*A*	*D*⋯*A*	*D*—H⋯*A*
O5—H5*A*⋯O4^i^	0.86	1.90	2.728 (2)	161
O5—H5*B*⋯O2	0.86	1.95	2.762 (2)	156
O6—H6*A*⋯O3^ii^	0.89	2.18	2.819 (5)	128

Symmetry codes: (i) 
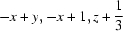
; (ii) 
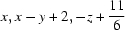
.

**Fig. 1.**
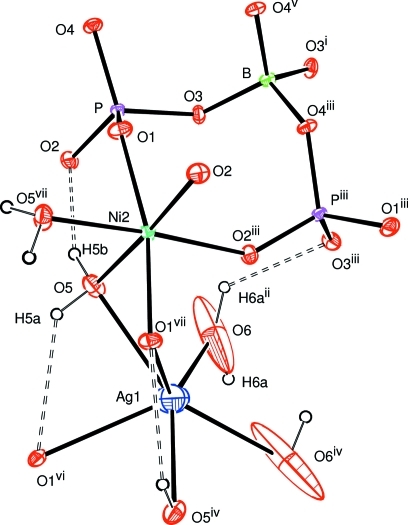
Partial plot of (Ag0.57Ni0.22)Ni(H2O)2[BP2O8],0.67(H2O) crystal structure showing plyhedra linkage. Displacement ellipsoids are drawn at the 50% probability level. Symmetry codes: (i) -y + 1, -x + 1, -z + 13/6; (ii) y - 1, -x + y, z + 1/6; (iii) y - 1, x, -z + 5/3; (iv) x, x-y + 1, -z + 11/6; (v) -x + y - 1, y, -z + 3/2; (vi) -x, -x + y, -z + 4/3; (vii) y, x + 1, -z + 5/3; (viii) x-y + 1, -y + 2, -z + 2.

**Fig. 2.**
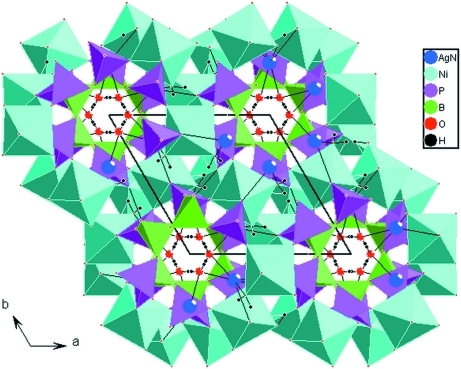
Projection view of the (Ag0.57Ni0.22)Ni(H2O)2[BP2O8],0.67(H2O) framework structure showing tunnel running along c direction where water molecules are located.

## supplementary crystallographic information

### Comment

A large group of borophosphates is known with a molar ratio of B:*P* = 1:2 and helical structure type, which consist of loop branched chain anions built from tetrahedral BO_4_ and PO_4_ units (Kniep *et al.*, 1998; Ewald *et al.*, 2007; Lin *et al.* 2008).

The aim of this work is the synthesis and the crystal structure of a new borophosphate-hydrate (Ag_0.57_Ni_0.22_)Ni(H_2_O)_2_[BP_2_O_8_],0.67(H_2_O), which is isotypic with analogue nickel borophosphates A(I)M(H_2_O)_2_[BP_2_O_8_] H_2_O (A(I)=Li, Na, K, NH_4_^+^; *M*(II)=Mg, Mn, Fe, Co, Ni, Cu, Zn, Cd) (Zouihri *et al.*, 2011; Menezes *et al.*, 2008).

The anionic partial structure of the title compound contains one–dimensional infinite helices, [BP_2_O_8_]^3–^, which are wound around 6_5_ axis. It is built up from alternate borate (BO_4_) and phosphate (PO_4_) tetrahedra, forming a spiral ribbon. The Ni^2+^ cation is six fold coordinated by four oxygen atoms originating from four phosphate groups and two water molecules as shown in Fig.1. The ribbons are interconnected through slightly distorted NiO_4_(H_2_O)_2_ octahedra. The resulting 3-D framework shows hexagonal tunnels running along c direction where water molecules are located (Fig.2).

The +I, +II, +III and +V oxidation states of the Ag, Ni, B and P atoms were confirmed by bond valence sum calculations (Brown & Altermatt, 1985). The calculated values for the Ag^+^, Ni^II+^, B^III+^ and P^V+^ ions are as expected, *viz*. 1.06, 1.89, 3.06 and 4.97, respectively.

The main difference between the structure of this compound and that of his counterpart AgMg(H_2_O)_2_[BP_2_O_8_],H_2_O lies in the filling ratio of the Wyckoff positions 6a and 6b in tunnels. Indeed, in this work, the refinement of the occupancy rate of the following sites (symmetry 2) 6a (x, 0, 0) and 6b (x, 2x, 3/4) (space group P6_5_22) shows that the first is occupied by water at 67% and the second is partially occupied by 56.6% of Ag^+^ and 21.6% of Ni^2+^ for a total of 78%. While in the case of AgMg(H_2_O)_2_[BP_2_O_8_],H_2_O structure these two sites are completely occupied by H_2_O and Ag^+^ respectively.

The structure is stabilized by O—H···O hydrogen bonds between water molecules and O atoms that are part of the helices (Fig.1 and Table 1).

### Experimental

The compound was hydrothermally synthesized at 453 K for 7 days in a 25 ml Teflon-lined steel autoclave from the mixture of NiCO_3_, H_3_BO_3_, H_3_PO_4_ (85%), AgNO_3_ and 5 mL of distilled water in the molar ratio of 1:4:6:1:165. The brilliant colourless octahedral crystals were recovered and washed with hot water, then dried in air.

Except for boron and hydrogen the presence of the elements were additionally confirmed by EDAX measurements.

### Refinement

The refinement with the sites of Ag and O6 fully occupied led to R = 0.06, Rw = 0.20 and two peaks in the Fourier difference map +4.7 and -3.4 at 0.18 Å from Ni and at 0.35 Å from Ag  respectively. While the refinement of the occupancy rate of Ag(0.350 (2)) and O6(0.34 (1)) leads to R = 0.027, Rw = 0.07 and two peaks +1.21 and -0.66 at 0.65Å and 0.52Å from Ag. In the final refinement cycles, the occupancy of O6 was fixed to 0.667. However, the large atomic displacement parameter for this atom indicate disorder or atomic movement along the partially empty tunnel.

The electrical neutrality of the molecule led us to put some nickel in the site of Ag. The refinement of this model leads to the ratio Ag/Ni about 0.47. In the  site occupied by a mixture of Ag^+^ and Ni^2+^, the cations are constrained to have the same positional and displacement parameters and the sum of the occupancy rate is restrained to fit the charge balance. The highest peak and the minimum peak in the difference map are at 0.66 Å and 0.52 Å respectively from Ag1 atom. The O-bound H atoms were initially located in a difference map and refined with O—H distance restraints of 0.86 (1). In the last cycles they were refined in the riding model approximation with *U*_iso_(H) set to 1.5*U*_eq_(O). The 779 Friedel opposite reflections are not merged.

### Crystal data

**Table:**

(Ag_0.57_Ni_0.22_)Ni(H_2_O)_2_[BP_2_O_8_]·0.67H_2_O	*D*_x_ = 3.145 Mg m^−^^3^
*M**_r_* = 381.35	Mo *K*α radiation, λ = 0.71073 Å
Hexagonal, *P*6_5_22	Cell parameters from 1912 reflections
Hall symbol: P 65 2 ( 0 0 1)	θ = 3.6–36.9°
*a* = 9.3848 (6) Å	µ = 4.69 mm^−^^1^
*c* = 15.8411 (18) Å	*T* = 296 K
*V* = 1208.28 (18) Å^3^	Prism, colourless
*Z* = 6	0.18 × 0.12 × 0.11 mm
*F*(000) = 1118	

### Data collection

**Table:**

Bruker APEXII CCD detector diffractometer	2043 independent reflections
Radiation source: fine-focus sealed tube	1912 reflections with *I* > 2σ(*I*)
graphite	*R*_int_ = 0.037
ω and φ scans	θ_max_ = 36.9°, θ_min_ = 3.6°
Absorption correction: multi-scan (*SADABS*; Sheldrick, 1999)	*h* = −14→15
*T*_min_ = 0.705, *T*_max_ = 0.741	*k* = −13→15
16974 measured reflections	*l* = −25→26

### Refinement

**Table:**

Refinement on *F*^2^	Secondary atom site location: difference Fourier map
Least-squares matrix: full	Hydrogen site location: difference Fourier map
*R*[*F*^2^ > 2σ(*F*^2^)] = 0.028	H-atom parameters constrained
*wR*(*F*^2^) = 0.071	*w* = 1/[σ^2^(*F*_o_^2^) + (0.043*P*)^2^ + 0.3826*P*] where *P* = (*F*_o_^2^ + 2*F*_c_^2^)/3
*S* = 1.08	(Δ/σ)_max_ = 0.001
2043 reflections	Δρ_max_ = 1.23 e Å^−^^3^
77 parameters	Δρ_min_ = −0.59 e Å^−^^3^
1 restraint	Absolute structure: Flack (1983), 777 Friedel pairs
Primary atom site location: structure-invariant direct methods	Flack parameter: 0.006 (15)

### Special details

**Table:**

Geometry. All s.u.'s (except the s.u. in the dihedral angle between two l.s. planes) are estimated using the full covariance matrix. The cell s.u.'s are taken into account individually in the estimation of s.u.'s in distances, angles and torsion angles; correlations between s.u.'s in cell parameters are only used when they are defined by crystal symmetry. An approximate (isotropic) treatment of cell s.u.'s is used for estimating s.u.'s involving l.s. planes.
Refinement. Refinement of *F*^2^ against all reflections. The weighted *R*-factor *wR* and goodness of fit *S* are based on *F*^2^, conventional *R*-factors *R* are based on *F*, with *F* set to zero for negative *F*^2^. The threshold expression of *F*^2^ > 2σ(*F*^2^) is used only for calculating *R*-factors(gt) *etc*. and is not relevant to the choice of reflections for refinement. *R*-factors based on *F*^2^ are statistically about twice as large as those based on *F*, and *R*- factors based on all data will be even larger.

### Fractional atomic coordinates and isotropic or equivalent isotropic displacement parameters (Å^2^)

**Table:**

	*x*	*y*	*z*	*U*_iso_*/*U*_eq_	Occ. (<1)
Ag1	0.18588 (3)	0.81412 (3)	1.0833	0.03293 (14)	0.567 (2)
Ni1	0.18588 (3)	0.81412 (3)	1.0833	0.03293 (14)	0.2167 (12)
Ni2	0.10776 (4)	0.553882 (19)	0.9167	0.00726 (7)	
P	0.17053 (5)	0.78027 (5)	0.75196 (3)	0.00600 (8)	
B	−0.15242 (15)	0.6952 (3)	0.7500	0.0068 (4)	
O1	0.13668 (19)	0.61873 (17)	0.79010 (8)	0.0111 (2)	
O2	0.31964 (17)	0.93224 (18)	0.78587 (8)	0.0111 (2)	
O3	0.02186 (17)	0.80809 (17)	0.76714 (8)	0.0088 (2)	
O4	0.18247 (16)	0.76331 (16)	0.65399 (8)	0.0084 (2)	
O5	0.29029 (19)	0.79869 (19)	0.94467 (10)	0.0150 (3)	
H5A	0.3751	0.8066	0.9698	0.022*	
H5B	0.3290	0.8571	0.8997	0.022*	
O6	0.1235 (7)	1.0000	1.0000	0.135 (5)	0.67
H6A	0.1238	1.0524	1.0471	0.203*	0.67

### Atomic displacement parameters (Å^2^)

**Table:**

	*U*^11^	*U*^22^	*U*^33^	*U*^12^	*U*^13^	*U*^23^
Ag1	0.0385 (2)	0.0385 (2)	0.0261 (2)	0.0225 (2)	−0.00270 (16)	−0.00270 (16)
Ni1	0.0385 (2)	0.0385 (2)	0.0261 (2)	0.0225 (2)	−0.00270 (16)	−0.00270 (16)
Ni2	0.00732 (13)	0.00740 (10)	0.00704 (11)	0.00366 (6)	0.000	0.00101 (9)
P	0.00595 (16)	0.00698 (16)	0.00472 (14)	0.00296 (13)	0.00006 (13)	0.00092 (13)
B	0.0085 (7)	0.0070 (9)	0.0046 (8)	0.0035 (4)	0.0006 (7)	0.000
O1	0.0162 (6)	0.0098 (5)	0.0080 (5)	0.0069 (5)	0.0009 (4)	0.0024 (4)
O2	0.0068 (5)	0.0109 (6)	0.0104 (5)	0.0004 (4)	−0.0005 (4)	−0.0007 (4)
O3	0.0053 (5)	0.0102 (5)	0.0107 (5)	0.0037 (4)	−0.0001 (4)	−0.0022 (4)
O4	0.0111 (5)	0.0107 (5)	0.0051 (4)	0.0066 (5)	0.0010 (4)	0.0016 (4)
O5	0.0112 (6)	0.0138 (6)	0.0159 (6)	0.0033 (5)	−0.0028 (5)	0.0037 (5)
O6	0.045 (2)	0.112 (7)	0.271 (12)	0.056 (3)	−0.077 (4)	−0.154 (8)

### Geometric parameters (Å, °)

**Table:**

Ag1—O5	2.4389 (17)	Ni2—O5	2.1152 (16)
Ag1—O5^i^	2.4389 (17)	Ni2—O5^ii^	2.1152 (16)
Ag1—O6	2.480 (4)	P—O1	1.5108 (14)
Ag1—O6^i^	2.480 (4)	P—O2	1.5117 (15)
Ag1—O1^ii^	2.6569 (15)	P—O3	1.5611 (14)
Ag1—O1^iii^	2.6569 (15)	P—O4	1.5698 (13)
Ni2—O2^iv^	2.0610 (14)	B—O3^vi^	1.462 (2)
Ni2—O2^v^	2.0610 (14)	B—O3	1.462 (2)
Ni2—O1^ii^	2.0733 (13)	B—O4^vii^	1.477 (2)
Ni2—O1	2.0733 (13)	B—O4^iv^	1.477 (2)
			
O5—Ag1—O5^i^	132.92 (8)	O2^v^—Ni2—O5^ii^	88.46 (7)
O5—Ag1—O6	78.79 (10)	O1^ii^—Ni2—O5^ii^	88.16 (6)
O5^i^—Ag1—O6	147.60 (8)	O1—Ni2—O5^ii^	82.71 (6)
O5—Ag1—O6^i^	147.60 (8)	O5—Ni2—O5^ii^	90.93 (10)
O5^i^—Ag1—O6^i^	78.79 (10)	O1—P—O2	115.82 (8)
O6—Ag1—O6^i^	71.1 (2)	O1—P—O3	110.43 (8)
O2^iv^—Ni2—O2^v^	92.40 (9)	O2—P—O3	105.70 (8)
O2^iv^—Ni2—O1^ii^	87.94 (5)	O1—P—O4	106.47 (7)
O2^v^—Ni2—O1^ii^	101.12 (6)	O2—P—O4	111.57 (8)
O2^iv^—Ni2—O1	101.12 (6)	O3—P—O4	106.52 (7)
O2^v^—Ni2—O1	87.94 (5)	O3^vi^—B—O3	102.25 (19)
O1^ii^—Ni2—O1	166.98 (9)	O3^vi^—B—O4^vii^	112.61 (8)
O2^iv^—Ni2—O5	88.46 (7)	O3—B—O4^vii^	113.91 (7)
O2^v^—Ni2—O5	176.10 (6)	O3^vi^—B—O4^iv^	113.91 (7)
O1^ii^—Ni2—O5	82.71 (6)	O3—B—O4^iv^	112.61 (8)
O1—Ni2—O5	88.16 (6)	O4^vii^—B—O4^iv^	102.02 (18)
O2^iv^—Ni2—O5^ii^	176.10 (6)		

Symmetry codes: (i) −*y*+1, −*x*+1, −*z*+13/6; (ii) *x*, *x*−*y*+1, −*z*+11/6; (iii) −*x*+*y*, −*x*+1, *z*+1/3; (iv) *y*−1, −*x*+*y*, *z*+1/6; (v) *y*−1, *x*, −*z*+5/3; (vi) −*x*+*y*−1, *y*, −*z*+3/2; (vii) −*x*, −*x*+*y*, −*z*+4/3.

### Hydrogen-bond geometry (Å, °)

**Table:**

*D*—H···*A*	*D*—H	H···*A*	*D*···*A*	*D*—H···*A*
O5—H5A···O4^iii^	0.86	1.90	2.728 (2)	161.
O5—H5B···O2	0.86	1.95	2.762 (2)	156.
O6—H6A···O3^viii^	0.89	2.18	2.819 (5)	128.

Symmetry codes: (iii) −*x*+*y*, −*x*+1, *z*+1/3; (viii) *x*, *x*−*y*+2, −*z*+11/6.
